# De Novo Growth Zone Formation from Fission Yeast Spheroplasts

**DOI:** 10.1371/journal.pone.0027977

**Published:** 2011-12-15

**Authors:** Felice D. Kelly, Paul Nurse

**Affiliations:** 1 The Rockefeller University, New York, New York, United States of America; 2 The Francis Crick Institute, London, United Kingdom; University of Cambridge, United Kingdom

## Abstract

Eukaryotic cells often form polarized growth zones in response to internal or external cues. To understand the establishment of growth zones with specific dimensions we used fission yeast, which grows as a rod-shaped cell of near-constant width from growth zones located at the cell tips. Removing the cell wall creates a round spheroplast with a disorganized cytoskeleton and depolarized growth proteins. As spheroplasts recover, new growth zones form that resemble normal growing cell tips in shape and width, and polarized growth resumes. Regulators of the GTPase Cdc42, which control width in exponentially growing cells, also control spheroplast growth zone width. During recovery the Cdc42 scaffold Scd2 forms a polarized patch in the rounded spheroplast, demonstrating that a growth zone protein can organize independent of cell shape. Rga4, a Cdc42 GTPase activating protein (GAP) that is excluded from cell tips, is initially distributed throughout the spheroplast membrane, but is excluded from the growth zone after a stable patch of Scd2 forms. These results provide evidence that growth zones with normal width and protein localization can form de novo through sequential organization of cellular domains, and that the size of these growth zones is genetically controlled, independent of preexisting cell shape.

## Introduction

Cells grow with a wide range of sizes and morphologies. Fission yeast exhibits a constant cylindrical shape and a near-constant cell width throughout the vegetative cell cycle, with growth localized to the cell tips [1]. This stable cell shape has been instrumental in exploring genetic determinants of morphology, but the cells' persistent shape makes it difficult to determine which of the many proteins that influence growth are primarily responsible for organizing the shape of the cell. One approach to studying the establishment of the rod shape has been to physically constrain cells in polymer molds of different shapes and see how the cytoskeleton and cell growth patterns respond [2], [3]. These studies have shown that microtubule contact with the cell cortex can establish new growth zones at novel sites in a bent cell, as predicted by supernumerary growth zone formation in cells with shortened microtubules [4].

Here we took a different approach: removing the cell wall to create depolarized, osmotically sensitive spheroplasts and then studying the recovery of normal cell shape. Fission yeast, unlike budding yeast, can recover in liquid media from complete cell wall digestion [5]. Previous work on fission yeast spheroplast recovery has focused primarily on the first few hours after spheroplast formation, when the cell wall initially regrows around the entire spheroplast [6], [7]. This initial cell wall regrowth is partly polarized, involves the polarization of actin [8], and is inhibited by cytochalasin D, which disrupts actin polymerization [5]. Later, as cell wall recovery proceeds to cover the entire surface of the cell, actin becomes depolarized [8]. The spheroplast at this stage is round or ellipsoid, has regrown the cell wall, and is again resistant to rupture by osmotic stress.

## Results and Discussion

### Depolarized spheroplasts can re-form a normal growth zone

**Figure 1:**
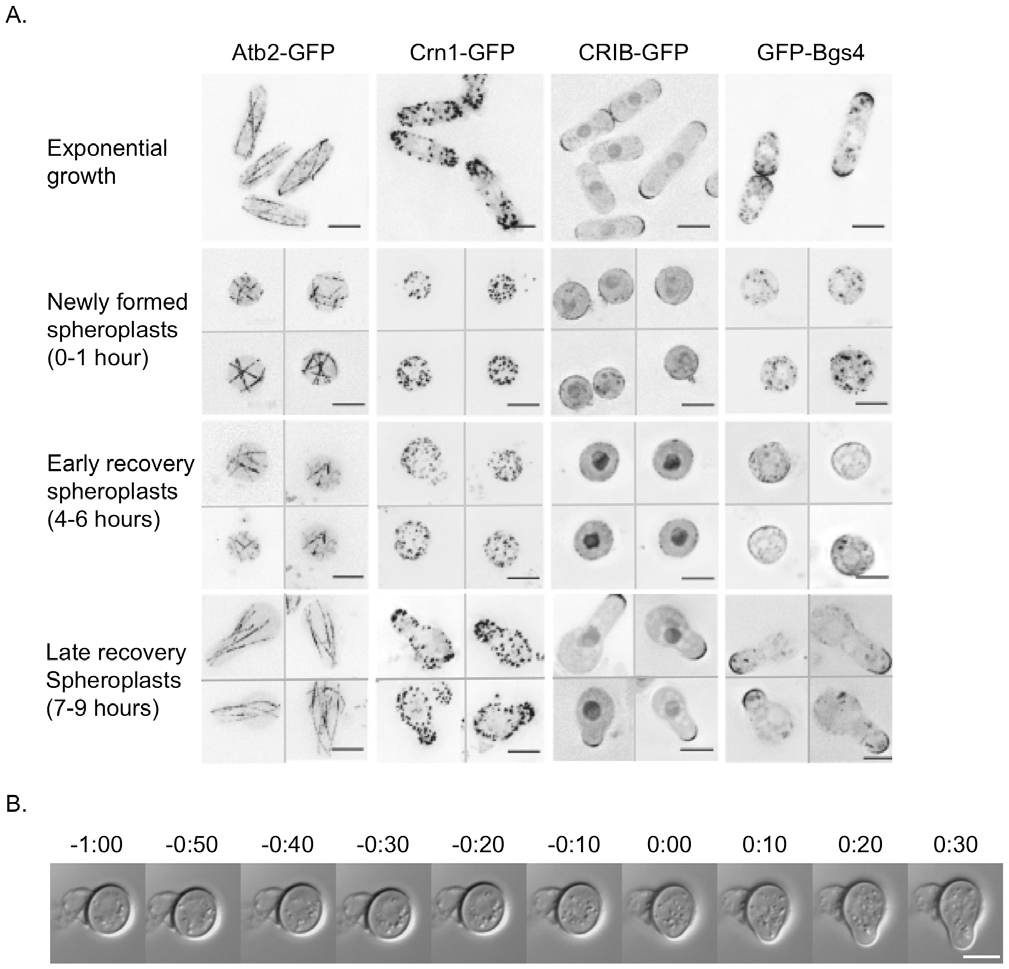
Depolarized spheroplasts can re-form a normal growth zone. **A.** Protein localization during spheroplast recovery; exponentially growing cells (top row) and cells at indicated times after spheroplast formation. All images show representative cells, and have been deconvolved, with inverted look-up tables (LUTs). For Atb2-GFP and Crn1-GFP, the images are maximum projections of Z-series that spanned the cell width. For GFP-Bgs4 and CRIB-GFP, the images show a single middle plane. Time is hours after spheroplast formation. For spheroplast recovery panels, different images in each panel are separated by a thin grey line. **B.** Time-lapse images of a single recovering spheroplast forming a growth zone. Time is in hours:minutes from spheroplast polarization. DIC image, best focal plane for each timepoint from a Z-series of 6 µm. All scale bars represent 5 µm.

To understand how the spheroplast regains its rod shape, we observed the complete progression of spheroplast recovery using a fluorescently tagged protein that marks microtubules (Atb2-GFP [9]), and three proteins that mark sites of active growth: an actin patch protein (Crn1-GFP [10]), a cell wall synthase (GFP-Bgs4 [11]), and a sensor for activated Cdc42 (CRIB-GFP [12]). In exponentially growing cells, microtubules are arranged in three to five bundles along the length of the cell [13], and the growth proteins localize to growing cell tips. In newly formed spheroplasts that were not surrounded by a cell wall, the parallel organization of microtubules was disrupted, and the growth proteins that normally localize to cell tips were distributed throughout the cells (Fig. 1A, newly formed spheroplasts). These proteins mark the cytoskeleton and some of the major pathways specifying growth, and their localizations were lost when cell shape was disrupted, likely erasing the history of organized growth within these cells.

To track the regrowth of the cell wall, we monitored spheroplast sensitivity to osmotic shock. When newly formed spheroplasts were moved to media that was not supplemented with sorbitol (or another osmoticum), they immediately ruptured. But after 5 hours' recovery, spheroplasts survived in non-supplemented media, indicating that the cell wall had regrown. Within the walled, round cells microtubules and growth proteins were still disorganized (Fig. 1A, early recovery spheroplasts). These early recovery spheroplasts then either divided or radically changed shape to form a new growth zone. This new growth zone resembled the tip growth of exponentially growing cells, with microtubules again arranged along the long axis of the cell, and the growth proteins localized to a distinct domain at the cell tip (Fig. 1A, late recovery spheroplasts). To better understand the formation of a new growth zone we observed the transition from early to late recovery spheroplast by time-lapse imaging (Fig. 1B). Early recovery spheroplasts were round, and did not grow preferentially in any direction, as shown for a single cell in Fig. 1B, panels 1–6. Then the growth pattern suddenly changed, and the cells formed a distinct localized growth zone and began polarized growth, as shown in Fig. 1B, panels 7–10.

These data show that spheroplasts are depolarized and that cells recover by first regrowing the cell wall and then forming a distinct growth zone, which leads to the reestablishment of the rod shape of the cell. The transition to polarized growth is accompanied by a major reorganization of the tubulin and actin cytoskeletons, cell wall production, and Cdc42 activation.

### The Cdc42 pathway affects the width of a growth zone formed de novo

**Figure 2:**
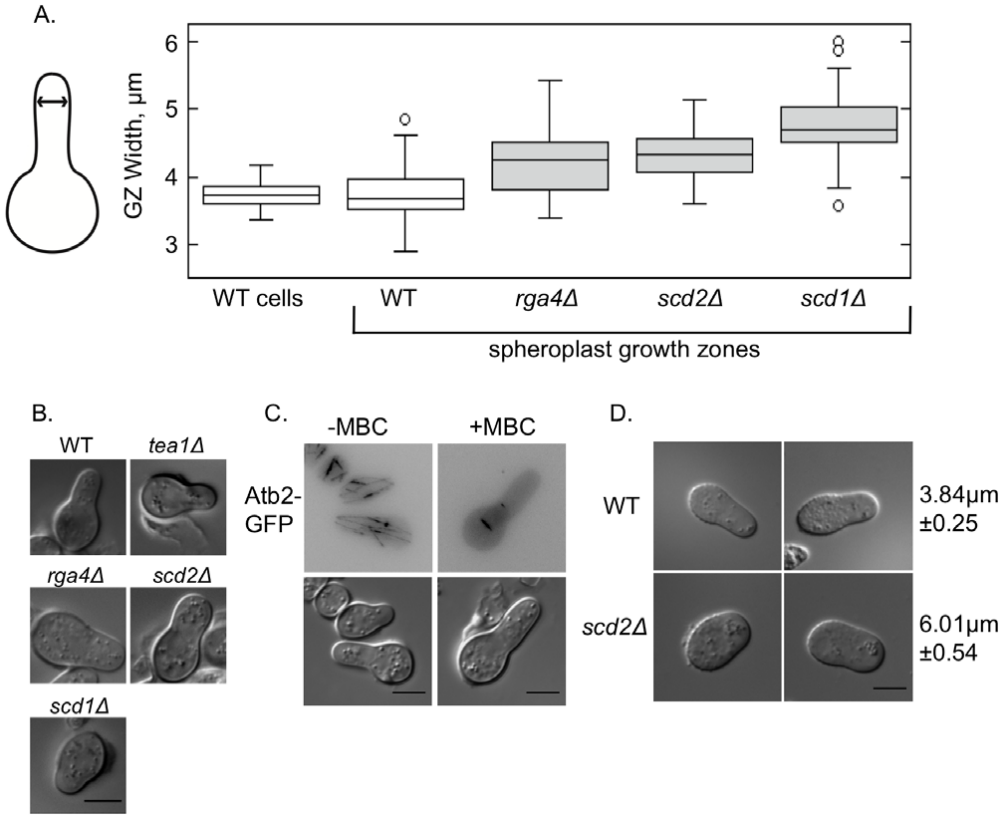
The Cdc42 pathway affects the width of growth zones formed de novo. **A.** The width of a spheroplast growth zone is the same as the width of a non-spheroplasted cell, but is altered by the mutation of genes that control cell morphology. Cell widths are represented in box-and-whiskers plots. All widths except for the first point are from late recovery spheroplasts, measured as indicated in the cartoon, 7–8 h after spheroplast formation; 50 cells were measured for each genotype or treatment. **B.** Late recovery spheroplasts of the indicated genotypes. DIC microscopy. **C.** Addition of 50 µM MBC depolymerizes microtubules, leaving only short stubs, but does not inhibit spheroplast growth zone formation. Top row: Atb2-GFP marks microtubules, and is shown as a maximum projection of Z-series that spanned the cell width, LUTs inverted. Bottom row: Cell shape by DIC. **D.** Spore germination forms a de novo growth zone, similar to spheroplast recovery. Right column shows growth zone widths, 50 cells measured for each genotype, displayed ±S.D. Wild-type exponentially growing cell width = 3.88 µm±0.15; *scd2Δ* exponentially growing cell width = 5.34 µm±0.25. DIC microscopy. All scale bars represent 5 µm.

The de novo formation of a distinct growth zone provides a tool to ask whether the proteins that influence the morphology of an exponentially growing cell are required for the formation of a new growth zone. To investigate how closely a de novo growth zone resembled an exponentially growing cell tip, we tested to see if the same genes that affect cell morphology affect spheroplast recovery. We found that in polarized spheroplasts, the newly formed growth zone was the same width as an exponentially growing cell (Fig. 2A). Blocking cells at the G2/M transition using the temperature sensitive *cdc25-22* cell-cycle mutant [14] did not prevent growth zone formation (data not shown), and the newly formed growth zones were the same width as blocked control cells (*cdc25-22* blocked control cells' average width = 4.31 µm±0.67 vs. *cdc25-22* recovering spheroplasts' average growth zone width = 4.29 µm±0.42). When the *cdc25-22* mutant is blocked in the cell cycle, both spheroplasted and non-spheroplasted cells became wider, but there was no significant difference between spheroplasted and non-spheroplasted cells' widths.

The process of spheroplast polarization may be similar to spontaneous symmetry breaking in budding yeast bud-site-selection mutants. In cells where the bud-site-selection mechanisms have been deleted, stochastic Cdc42 activation determines the new bud site [15], [16]. In fission yeast, Cdc42 regulators influence cell morphology and are required for the maintenance of normal cell width [17], [18], [19], [20]. To determine if these genes also influence spheroplast recovery, we compared the late recovery spheroplast growth zones of deletion mutants and wild-type cells. We focused on deletion mutants of three genes that are closely related to the control of the small GTPase Cdc42: the genes encoding the GTPase activating protein (GAP) *rga4* [2], [3], the GTPase exchange factor (GEF) *scd1* [19], [21], and the scaffold protein *scd2* [22]. Each of these deletion mutants was able to form growth zones de novo from spheroplasts, indicating that these genes are not required for growth polarization. In each case the new growth zones were wider than wild-type growth zones, just as these deletion mutants are wider in exponential growth (Figure 2A, B). Therefore we conclude that the Cdc42 pathway also influences the size of the growth zone in spheroplasts, which have no history of a specific cell width derived from the cell wall.

Microtubules and the microtubule-delivered morphology protein Tea1 have been implicated in the formation of growth zones and in the straight-line growth of cells [23]. Though Tea1 is required for the initiation of bipolar growth, it was not required for growth zone formation in the spheroplast (Fig. 2B). We investigated if a growth zone could be formed de novo in the absence of microtubules. Spheroplasts were treated with carbendazim (MBC), which inhibits microtubule polymerization [24], and Atb2-GFP was used to monitor microtubules. In cells treated with high levels of MBC (50 µg/mL), we observed no full-length microtubules reaching the cell cortex, though some short stubs remained (Fig. 2C). Even in these conditions spheroplasts were able to polarize growth, showing that full-length microtubules are not required for the establishment of a new growth zone.

Fission yeast also polarizes growth from a round spore after mating. When spores form after meiosis, each spore membrane is newly formed within the ascus, and so will not carry marks of previous polarized growth [25]. When these spores are exposed to favorable growth conditions they begin polarized growth with a germination tube. The width of the wild-type germination tube was similar to the width of an exponentially growing wild-type cell (Fig. 2D). To look at whether germination tube width and growth zone width are controlled by the same genes, we generated a homozygous diploid of the deletion mutant *scd2Δ* and induced sporulation (Fig. 2D). Germinating *scd2Δ* spores produced wider germination tubes than those from wild-type cells, demonstrating that germination tube width is influenced by the deletion of *scd2*, as is growth zone size. This provides further evidence that the mechanism establishing the width of a growth zone does not depend on history, and that the genes that control cell width during normal growth also control de novo growth zone size. The *scd2Δ* germination tube was considerably wider than the *scd2Δ* recovering spheroplast growth zone, perhaps indicating some mechanistic differences between germination and spheroplast recovery.

### Cdc42 regulators do not carry the memory of the growth zone through spheroplasting

**Figure 3:**
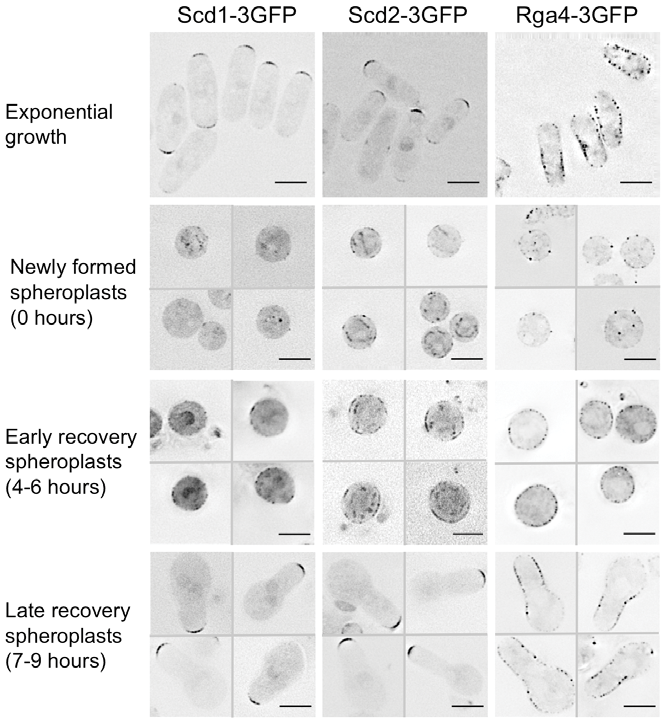
Cdc42 regulators do not carry the memory of the growth zone through spheroplasting. Protein localization during spheroplast recovery; images show exponentially growing cells (top row) and cells at indicated times after spheroplast formation. All images show single, middle planes from Z-stacks of representative cells, and have been deconvolved, with inverted LUTs. For spheroplast recovery panels, different images in each panel are separated by a thin grey line. Scale bars represent 5 µm.

Since the regulators of Cdc42 are important for establishing the width of the growth zone in recovering spheroplasts and exponentially growing cells, we investigated how they were localized during spheroplast formation and recovery. These proteins localize to the growth zone in the exponentially growing cell, and so could carry the shape and size of the growth zone through spheroplast formation and recovery by forming a persistent patch. As suggested from previously published data [17], [26], [27], Scd1-3GFP and Scd2-3GFP localized to cell tips in exponentially growing cells while Rga4-3GFP localized to cell sides (Fig. 3, top row). But these localizations did not persist through spheroplast formation: Scd1-3GFP, Scd2-3GFP, and Rga4-3GFP were depolarized in newly formed spheroplasts (Fig. 3, newly formed spheroplasts). Even after the cell wall had regrown these proteins were not organized in exclusive domains (Fig. 3, early recovery spheroplasts). But when new growth zones formed and the cell returned to linear extension, their localization was restored to limited domains in the new growth zones (Fig. 3, late recovery spheroplasts). These results indicate that the proteins that determine cell morphology are disorganized when spheroplasts form and then reorganize as the cell returns to tip-localized growth.

### Domains of Cdc42 activation and inhibition are sequentially organized

**Figure 4:**
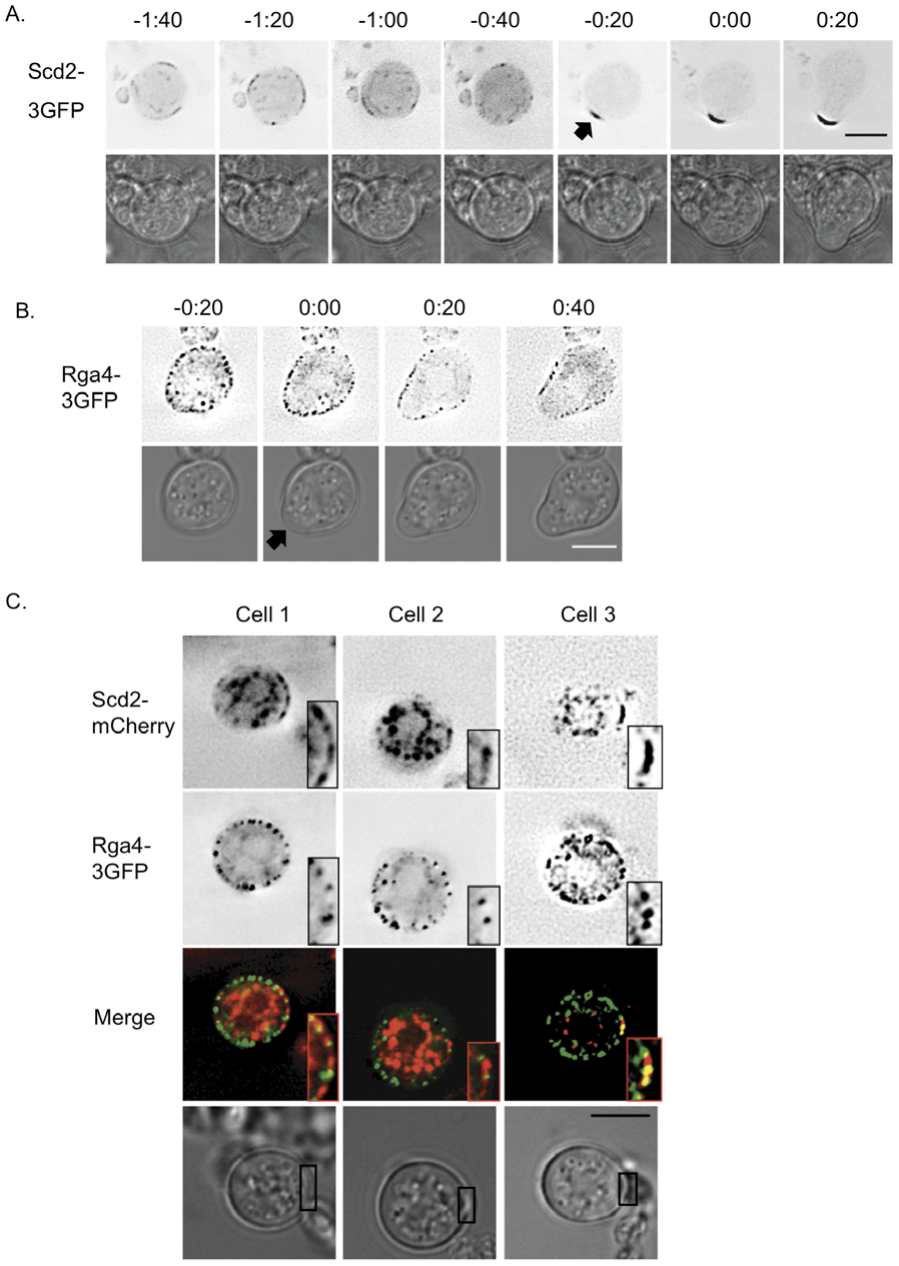
Protein polarization accompanies spheroplast recovery. **A.** Scd2 polarizes before cell shape changes. Time-lapse series of Scd2-3GFP in a spheroplast as it undergoes the transition to polarized growth. The black arrow indicates the appearance of a bright patch of Scd2-3GFP that formed before the shape of the spheroplast changed. We observed this patch formation preceding growth in all six Scd2-3GFP cells that polarized during time-lapse imaging, and in an additional twelve cells in a two-color time-lapse series with Scd2-mCherry. For an additional example see Figure S1A. **B.** Rga4 is excluded as the growth zone forms. Time-lapse series of Rga4-3GFP in a spheroplast as it undergoes the transition to polarized growth. Black arrow indicates the initiation and location of polarized growth. This clearing of Rga4 from the growth zone was observed in all 15 cells filmed. For an additional example see Figure S1B. In panels A and B, fluorescence images are single planes, best fluorescent signal, with inverted LUTs, and time is displayed in hours:minutes from spheroplast polarization. **C.** Scd2 organizes into a patch before Rga4 is excluded. Each of the three cells shown is an early recovery spheroplast, imaged just before polarized growth begins. Images are from the same time-point and Z-section for three different cells. Boxed insets show 2× magnification of the area of the Scd2-mCherry patch. In 10 of 12 cells observed, Scd2 formed a patch before Rga4 was cleared from the area, and in the remaining cells the order could not be determined. Black boxes on the brightfield image indicate the area of the Scd2 patch, shown in the boxed insets in the fluorescence images. Polarized growth, as determined by time-lapse, occurred at the same location as this Scd2 patch. For all panels, fluorescence images are single planes, inverted and deconvolved. Merge images are not inverted; green is Rga4-3GFP, red is Scd2-mCherry. All scale bars represent 5 µm.

To explore how the new polarized growth zone forms, we used time-lapse imaging of Scd2 and Rga4, two proteins that are involved in the specification of the size of the growth zone. Scd2-3GFP localizes to the growth zone in exponentially growing cells. In recovering spheroplasts this protein formed a distinct patch before cells had formed a growth zone (Fig. 4A and Figure S1A). This patch appeared and was eventually stabilized in the same location where the growth zone ultimately formed. In an exponentially growing cell, the shape of the cell might direct growth proteins to the cell tip, thus determining their localizations. But since Scd2 can form a protein patch in a round spheroplast, before the cell shape changes, its localization does not depend on the rod shape of the cell. Furthermore, in time-lapse analyses the new growth zone forms at the location of the Scd2 protein patch, potentially indicating a role for Cdc42 activation in forming the new growth zone. This is reminiscent of the process of symmetry breaking in budding yeast [28], [29], where the scaffold Bem1, which is a homolog of Scd2, is involved in stabilizing activated Cdc42 [16]. Rga4-3GFP, which localizes to the sides of normally growing cells ([17], Figure 3), was found throughout the surface of early recovery spheroplasts, but its localization changed as polarized growth began. As the cells changed shape, Rga4-3GFP was lost from the new growth zones (Fig. 4B and Figure S1B).

The time-lapse imaging of Rga4 and Scd2 in recovering spheroplasts led us to ask which of these growth zone components is organized first in the recovering spheroplast. Rga4 and Scd2 both contribute to the genetic control of cell width and localize to mutually exclusive zones in the cell, but the genesis of these zones is unknown. To investigate the order of recruitment we simultaneously monitored the localization of Rga4-3GFP and Scd2-mCherry in recovering spheroplasts. By combining data from the first time point where a distinct Scd2 patch was visible with time-lapse imaging to determine the eventual location of the growth zone, we could correlate Scd2 patch formation, Rga4 localization, and growth zone formation. We extracted data for fifteen double-labeled cells that polarized during the time-lapse. In twelve of these cells Scd2-mCherry formed a patch before the Rga4-3GFP localization had significantly changed, and in the remaining three it was not possible to determine which protein's localization changed first. Three cells of the former category from these experiments are shown in Fig. 4C. Since Scd2 forms a patch, and then Rga4 is excluded as growth begins, these observations provide evidence that the localization of Rga4 to cell sides in exponentially growing cells is mediated by exclusion from the growth zone. That exclusion could occur passively, if Rga4 were removed from the cell tip membrane by compensatory endocytosis, or it could occur actively, if a downstream effector of Cdc42 modified Rga4 and changed its membrane localization. Rga4 is known to be phosphorylated [12], and that phosphorylation or another modification could exclude it from areas where Cdc42 is activated.

Spheroplasts provide a system to investigate how growth zone proteins are directed to form their distinctive patches at the cell tips. Because the shape of a normally growing cell does not change during the cell cycle, it has been difficult to determine whether growth proteins are specifically localized to domains at the ends of the cell because of the preexisting shape of the cell, or whether they would self-organize to form those domains de novo. Our data show that growth proteins are completely delocalized when a spheroplast is formed, and remain delocalized even after cell wall formation in a round spheroplast. But then the spheroplast forms a new, distinct growth zone, which is the same width as a wild-type cell, and the same genes are important for determining its width. Cell-tip proteins become localized to that new growth zone, with Scd2 localization preceding growth zone formation. The data thus provide strong evidence that domains of growth can form with proper size and shape de novo through sequential organization of cellular domains, and do not rely solely on the preexisting shape of the cell.

## Materials and Methods

Yeast strains were grown as previously described [30]. For all experiments cells were grown in Edinburgh Minimal Media (EMM) with supplements (EMM4S) at 32°C, unless specified. For microscopy with fluorescent proteins, media was sterilized by filtration to reduce auto-fluorescence. All strains characterized in this study are listed in the supplementary strain table (Table S1). Deletion strain genotypes were verified by PCR across the kanamycin-resistance cassette. The deletion of genes and fluorescent tagging of proteins was performed by homologous recombination as described [8]. Strains expressing GFP-, mCherry-, and 3GFP-tagged proteins were analyzed to ensure that growth rate, cell size, and cell width were all similar to wild-type cells. All other strains were constructed by tetrad dissection, where applicable.

### Spheroplast generation

Spheroplasts were generated by enzymatic digestion of the cell wall, with slight alterations to a previously published protocol [8]. Cells were grown as an exponential culture in EMM4S to mid-log phase, then washed twice and resuspended in E-buffer+sorbitol (50 mM sodium citrate, 100 mM sodium phosphate buffer, +1.2 M Sorbitol, pH 5.6). To form spheroplasts, cells were incubated at 37°C with 0.2 mg/mL zymolyase 100T (Seikagaku Biobusiness) and 1.25 mg/mL lysing enzymes from trichoderma harzianum (Sigma). Cell wall digestion was monitored by estimating the percentage of spheroplasts, and cells were washed in EMM4S+1.2 M Sorbitol after >90% of cells appeared to be escaping the cell wall, ten to twenty minutes after enzyme addition. Cells were washed gently three times in EMM4S+1.2 M Sorbitol, and then allowed to recover in the same media, shaking, at either 32°C or 36.5°C. Early recovery spheroplasts form aggregates, and these were disrupted by brief sonication (<1 sec) using a Misonix Sonicator 3000 (Qsonica, Milton, CT, USA), medium tip, output strength 1.5.

For experiments with the temperature sensitive *cdc25-22* mutant, spheroplasts recovered at 36.5°C for 11 hours after spheroplasting before the growth zone width was measured. The control cells were also blocked for 11 hours. To depolymerize microtubules, newly formed spheroplasts were treated with carbendazim (MBC) for 7 hours, and then imaged live. The final MBC concentration was 50 µg/mL diluted from a freshly prepared 2 mg/mL stock solution in DMSO.

### Spheroplast imaging

For time-lapse imaging, spheroplasts were mounted on agarose pads (1.4% agarose in filtered EMM4S +1.2 M sorbitol). For time-lapse series greater than two hours, cover slips were sealed with VALAP (Vaseline, Lanolin, Paraffin; 1∶1∶1). For Figures 1B and 2, cells were imaged live using a Zeiss Axioplan 2 epifluorescence microscope equipped with a Zeiss αPlan Fluor ×100 N.A. 1.4, oil immersion objective, a CoolSNAP HQ camera (Roper Scientific) and Metamorph software (MDS Analytical Technologies), and analyzed in ImageJ (National Institutes of Health).

For Figures 1A, 3, and 4, cells were imaged live on the DeltaVision Spectris microscope (Applied Precision, Issaquah, WA, USA) composed of an Olympus IX71 wide-field inverted fluorescence microscope, an Olympus UPlanSApo ×100, N.A. 1.4, oil objective (Olympus, Center Valley, PA, USA), and a Photometrics CoolSNAP HQ camera (Roper Scientific). Images were processed by iterative constrained deconvolution using SoftWoRx (Applied Precision), and analyzed in ImageJ. Fluorescence images are shown with LUTs inverted for clearer display. The LUT inversion was done in ImageJ without altering the absolute intensity values.

Width measurements were made from photographs using the line tool in ImageJ. Cell width was measured on the growth zone, near the tip of the cell. In Figure 2A, cell widths are represented by box-and-whiskers plots. In these plots, the box represents the middle two quartiles of the data. The average of the data lies in the center of the box, and the median value is marked with a horizontal line across the box. The whiskers on the plot represent the outer quartiles of the distribution. If the range of the outer quartiles spans more than 1.5 times the size of the boxes then higher or lower values (outliers) are plotted as individual points.

### Diploid generation and spore germination

For spore germination, a homozygous h+/h+ diploid was generated by incubating a haploid h+ strain with 20 mg/mL MBC for 5 hours, and then plating the cells on rich media (YE4S) plates containing 5 mg/l phloxin B (Sigma). Diploids were selected by increased cell size, and then transformed with the plasmid pON177 that encodes the mat1-M mating factor [31]. These cells were then starved of nitrogen to induce sporulation. Spores were collected by digesting the ascus wall, washed, and resuspended in YE4S to induce germination. Germination was monitored, and germination tubes were photographed live 6 hours after return to YE4S media.

## Supporting Information

Figure S1 **Protein polarization accompanies spheroplast recovery; additional time-lapse images.** **A.** Scd2 polarizes before cell shape changes. Time-lapse series of Scd2-3GFP in a spheroplast as it undergoes the transition to polarized growth. **B.** Rga4 is excluded as the cell grows. Time-lapse series of Rga4-3GFP in a spheroplast as it undergoes the transition to polarized growth. Black arrow indicates the location of polarized growth. For A and B, fluorescence images are single planes, best fluorescent signal, with inverted LUTs. Time is in hours:minutes from spheroplast polarization, and the scale bars represent 5 µm.
(TIF)

Table S1 Strains used in this study.
(DOC)
