# Supplementary figures and images for: De Novo Growth Zone Formation from Fission Yeast Spheroplasts
Source: PLoS One. 2011 Dec 15;6(12):e27977. doi: 10.1371/journal.pone.0027977 (PMC3240611; doi:10.1371/journal.pone.0027977)

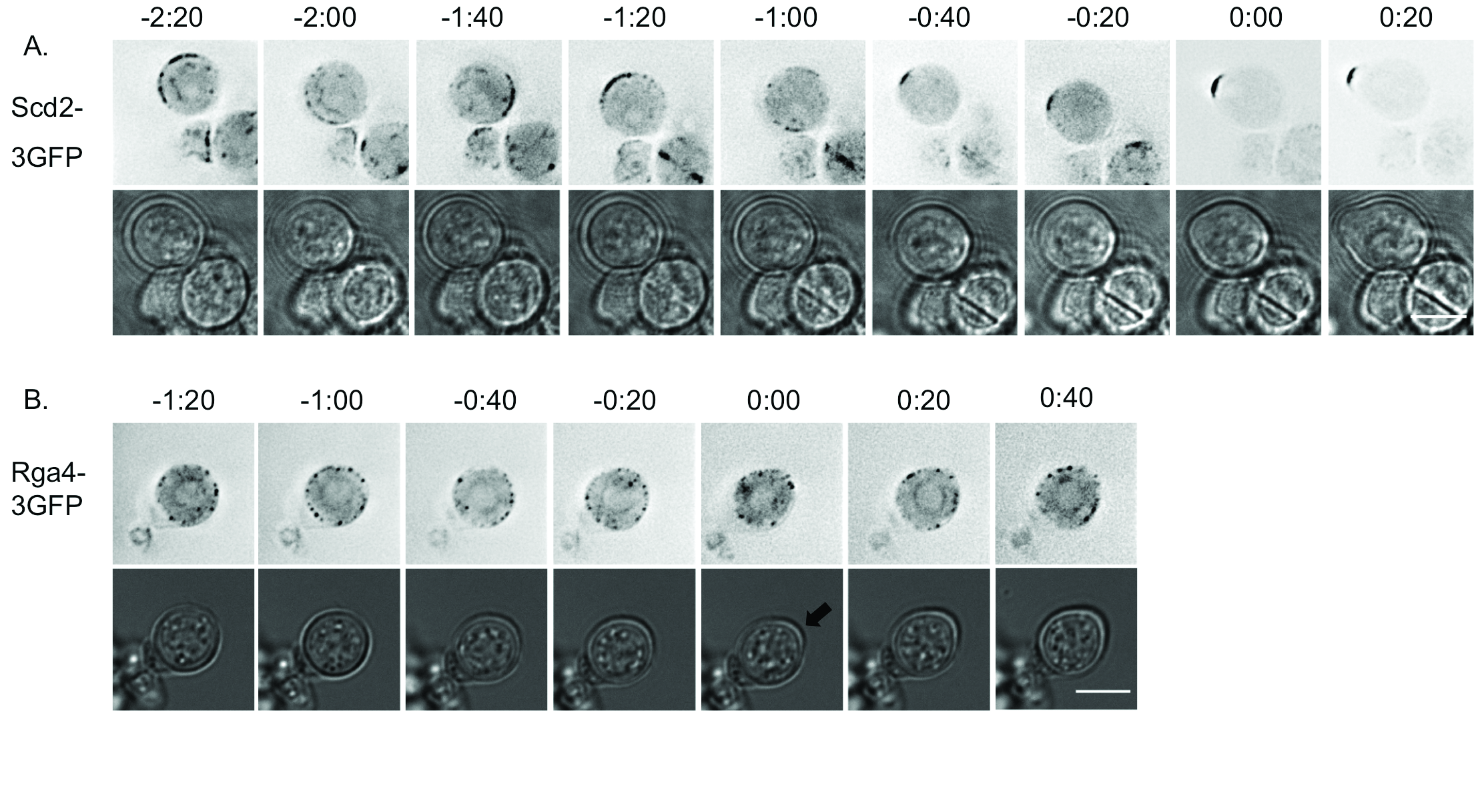

Supplement: Figure S1 — Protein polarization accompanies spheroplast recovery; additional time-lapse images. A. Scd2 polarizes before cell shape changes. Time-lapse series of Scd2-3GFP in a spheroplast as it undergoes the transition to polarized growth. B. Rga4 is excluded as the cell grows. Time-lapse series of Rga4-3GFP in a spheroplast as it undergoes the transition to polarized growth. Black arrow indicates the location of polarized growth. For A and B, fluorescence images are single planes, best fluorescent signal, with inverted LUTs. Time is in hours:minutes from spheroplast polarization, and the scale bars represent 5 µm. (TIF) [file pone.0027977.s001.tif]
