# Supplementary material for: De Novo Growth Zone Formation from Fission Yeast Spheroplasts
Source: PLoS One. 2011 Dec 15;6(12):e27977. doi: 10.1371/journal.pone.0027977 (PMC3240611; doi:10.1371/journal.pone.0027977)
Supplement: Table S1 — Strains used in this study. (DOC) [file pone.0027977.s002.doc]

Table S1: Strains used in this study

| Strain number | Genotype | Source |
| --- | --- | --- |
| PN556 | *ade6-216 ura4-D18 leu1-32 h+* | Lab collection |
| PN1733 | *tea1Δ::ura4+ ura4-D18 ade6-M210 h-* | [1] |
| PN10064 | *rga4Δ::kanMX6 ade6-M216 ura4-D18 leu1-32 h90* | (Kim et al., 2010) |
| PN10226 | *scd2Δ::kanMX6 ade6-M216 ura4-D18 leu1-32 h+* | (Kim et al., 2010) |
| PN10723 | *scd1Δ::kanMX6 ade6-M210 ura4-D18 leu1-32 h+* | Kelly and Nurse, in preparation |
| PN10727 | *rga4-3GFP::kanMX6 ura4-D18 leu1-32 ade6-M210 h+* | Kelly and Nurse, in preparation |
| PN10730 | *scd2-3GFP::KanMX6 h+* | Kelly and Nurse, in preparation |
| PN10732 | *scd1-3GFP::kanMX6 h+* | Kelly and Nurse, in preparation |
| CA5931 | *ura4-294::[P_pak1_+:ScGIC2 CRIB domain-3xGFP:ura4+] leu1-32* | [2] |
| PN4635 | *SV40-GFP-atb2-GFP::leu1+ leu1-32 ura4-D18 h-* | [3] |
| PN4251 | *crn1-GFP::kanMX6 ura4-D18 leu1-32 h+* | [4] |
| PN10355 | *bgs4*Δ*::ura4^+^ P_bgs4_^+^::GFP-bgs4^+^leu1-32 ura4-D18 hys3-D*h- | [5] |
| PN143 | *cdc25-22 h-* | Lab collection |
| PN10758 | *scd2::kanMX6/scd2::kanMX6 h+/h+* transformed with M factor plasmid pON177 | This study |
| PN10759 | *rga4-3GFP::kanMX6 scd2-mCherry::kanMX6* | This study |

**Supplemental References:**

S1. Kim, D.U., Hayles, J., Kim, D., Wood, V., Park, H.O., Won, M., Yoo, H.S., Duhig, T., Nam, M., Palmer, G., et al. (2010) Analysis of a genome-wide set of gene deletions in the fission yeast *Schizosaccharomyces pombe*. Nat. Biotechnol. 6: 617-623.
